# Production of Reproducible Filament Batches for the Fabrication of 3D Printed Oral Forms

**DOI:** 10.3390/pharmaceutics13040472

**Published:** 2021-03-31

**Authors:** Stéphane Roulon, Ian Soulairol, Valérie Lavastre, Nicolas Payre, Maxime Cazes, Laurent Delbreilh, Jean Alié

**Affiliations:** 1 Normandy University, UNIROUEN Normandie, INSA Rouen, CNRS, Group of Materials Physics, Av. Université, 76801 St Etienne du Rouvray CEDEX, France; 2 Solid State Characterization and 3D Printing Service, Sanofi R&D, 371 rue du Pr. Joseph Blayac, 34080 Montpellier CEDEX 4, France; valerie.lasvastre@sanofi.com (V.L.); Nicolas.Payre@Sanofi.com (N.P.); Maxime.Cazes@Sanofi.com (M.C.); 3 Department of Pharmacy, Nimes University Hospital, 30900 Nimes CEDEX 9, France; ian.soulairol@umontpellier.fr; 4 Department of galenic pharmacy and biomaterials, ENSCM, College of pharmacy, University of Montpellier, 34090 Montpellier CEDEX 5, France

**Keywords:** 3D printing, fused-filament fabrication, hot-melt extrusion, oral forms, filament, immediate release, pediatric

## Abstract

Patients need medications at a dosage suited to their physiological characteristics. Three-dimensional printing (3DP) technology by fused-filament fabrication (FFF) is a solution for manufacturing medication on demand. The aim of this work was to identify important parameters for the production of reproducible filament batches used by 3DP for oral formulations. Amiodarone hydrochloride, an antiarrhythmic and insoluble drug, was chosen as a model drug because of dosage adaptation need in children. Polyethylene oxide (PEO) filaments containing amiodarone hydrochloride were produced by hot-melt extrusion (HME). Different formulation storage conditions were investigated. For all formulations, the physical form of the drug following HME and fused-deposition modeling (FDM) 3D-printing processes were assessed using thermal analysis and X-ray powder diffraction (XRPD). Filament mechanical properties, linear mass density and surface roughness, were investigated by, respectively, 3-point bending, weighing, and scanning electron microscopy (SEM). Analysis results showed that the formulation storage condition before HME-modified filament linear mass density and, therefore, the oral forms masses from a batch to another. To obtain constant filament apparent density, it has been shown that a constant and reproducible drying condition is required to produce oral forms with constant mass.

## 1. Introduction

Tablets are the most widely used solid dosage form, accounting for 70% of all pharmaceutical preparations produced [1]. Tablets may be defined as solid pharmaceutical dosage forms containing drug substances prepared by either compression or molding process. It was in 1844 that the compression process was used for the manufacture of potassium bicarbonate tablets for therapeutic use [2]. Currently, tablets are formulated with a disintegrating agent, binders, glidant, lubricant and diluent [1].

Tablet dose flexibility is limited to the use of multiple units. Tablets can be scored to facilitate breaking, but there are risks related to dose uniformity and accuracy if breaking is not carried out properly [3]. Oral liquid formulations can be chosen for flexible patient dosages, but these have some disadvantages compared to tablets [4]. Liquid formulations are generally less stable and more expensive than tablets.

3D printing (3DP) is a process that has the potential to provide medicine with a dosage adapted to patient need [5]. 3DP is an additive manufacturing process that creates solid objects layer-by-layer. 3DP encompasses various techniques, such as stereolithography, inkjet, selective laser sintering and fused filament fabrication (FFF).

FFF 3D printers are low-cost, small machines [6]. The starting material is a filament containing thermoplastic polymer, which is melted to be deposited layer-by-layer to form an object. Today, FFF machines are used in a large number of industries as a rapid prototyping tool. In the pharmaceutical industry, FFF could be used as a personalized medicine production machine. The 3D-printed object corresponds to the oral form, and the filament is a continuous medicine formulation. It is possible to have different files in the 3D printer corresponding to different oral form sizes. The chosen size corresponds to a dosage adapted to the patient need [7]. This machine can be installed in hospitals, clinical centers and pharmacies to produce personalized oral forms on-demand [8].

For every oral form production, reproducibility between batches is a challenging topic. Environment, manufacturing process and formulation can play a role in batch reproducibility, which can cause undesirable attributes in oral form properties. This is why the European Medicines Agency (EMA) guidance give product quality attribute on oral forms mass and content uniformity [9]. Moreover, EMA indicates in guidance on process validation outline that formal process validation studies should be conducted on three consecutive batches [10]. The filament is the product driving oral form reproducibility. This is why the present study reports how to produce reproducible amiodarone filament batches.

In recent years, 3D printing in the pharmaceutical domain has developed significantly in different research groups on different aspects. Some teams work on the formulation [11,12,13], on the 3D-printed oral forms shapes [14], review possible 3D-printing use in the pharmaceutical industry [15] and study the patient acceptability [5]. Until today, no publication reports the study of filament batch reproducibility despite the fact it has been identified as a problem for the production of a constant mass of oral forms [16]. In this context, this publication reports the study of reproducible filament batches production for 3D-printed oral forms compounding.

It was demonstrated that the storage of the powder before the compression process has an influence on the physical properties of tablets prepared from them [17]. Powder interparticle forces can be modified by atmosphere relative humidity (RH). A high atmosphere humidity leads to higher interparticle forces due to capillary interactions [18,19]. This implies the formation of large agglomerates that are less breakable [20,21]. Pharmaceutical filaments are intermediate products produced from hot-melt extruded powders [22]. Filaments are used to produce oral forms; therefore, it is the product intended to be sent to hospitals and pharmacies. The environmental parameters such as humidity and temperature are parameters influencing the physical characteristics of the extrudate [23,24]. It is, therefore, essential to evaluate powder storage’s influence on filament physical properties. This is why different storage conditions were tested.

In this article, 3D-printed oral forms containing amiodarone hydrochloride have been developed for pediatric use. Amiodarone hydrochloride is an antiarrhythmic drug used to treat patients with cardiac arrhythmias. Some hospitals are currently having to prepare oral forms to adjust the dosage of amiodarone delivered. In fact, the use of medicines in children requires dosage adjustment according to the patient characteristics (weight, body surface, etc.). This is why the development of 3D-printed oral forms containing amiodarone hydrochloride can make it possible to produce oral forms on-demand and in a personalized way. In order to identify the influence of powder storage on batch reproducibility, six identical powder mixtures were prepared, analyzed, stocked in different conditions. These powders were used to produce filament batches to observe the influence of powder storage on filaments quality. Filament quality was investigated by weighing, thermal analysis, X-ray powder diffraction, scanning electron microscopy (SEM), and diameter measurement to obtain information regarding the linear mass density (LMD), thermal comportment, crystalline state, surface roughness and dimension variation. These analyses were also conducted on 3D-printed solid oral forms to observe the influence of the filament properties on oral form quality. A batch of 20 oral forms was produced in order to perform a mass uniformity measurement in accordance with the European Pharmacopoeia.

## 2. Materials and Methods

### 2.1. Materials

d-sorbitol (CE) was purchased from Carlo Erba. Polyethylene oxide, PEO(PolyoxN10, Dow Chemical, Midland, TX, USA), was donated. Glycerol (Glycerol, VWR, Radnor, PA, USA) was purchased. Colloidal anhydrous silica (Aerosil 200 Pharma, Evonik, Rheinfelden, DE, USA) was purchased. Amiodarone hydrochloride was donated from Sanofi, FR.

### 2.2. Method

#### 2.2.1. Blending

The polymer allowing the manufacture of the filaments is polyethylene oxide (PEO). PEO is a water-soluble thermoplastic polymer [25] that is known to be printable by the FFF process [26]. PEO provides good mechanical flexibility to the filament and is extrudable at temperatures lower than 100 °C [27]. Glycerol was selected for its plasticizing properties [28,29,30] in order to reduce the extrusion temperature and to obtain a flexible filament that can be used by the FFF 3D-printing process [31,32]. In this work, the selected filler chosen to produce a fast disintegration formulation was d-sorbitol. d-sorbitol is a filler with a high solubility [33]. This product has been used in 3D-printing formulations as a plasticizer in combination with PVA [34] or as a temporary plasticizer [35]. Colloidal anhydrous silica was used to obtain flow properties allowing an even feed of the powder into the extruder [36,37].

First, the thermoplastic polymer, PEO, was weighed then the liquid plasticizer (glycerol) was added by mixing/grinding in a mortar. Once the pre-blend was homogeneous, the active ingredient (amiodarone hydrochloride) was added to the previous mixture by mixing/grinding. The same process was used to add the filling (d-sorbitol) and the gliding agent (colloidal anhydrous silica).

PEO was chosen for its low melting temperature and the mechanical properties of the filaments obtained from this product [38]. When this extrudate was released at room temperature, it solidifies, which allows keeping a form of the filament. The plasticizer lowers the extrusion temperature and creates a more flexible filament [32].

d-sorbitol was used as a filler in the formulation for reducing the amount of thermoplastic polymer used. In a fast disintegration formulation, the polymer quantity is reduced to obtain better disintegration kinetic.

Colloidal anhydrous silica helps in free-flowing powder inside extrusion hopper by minimizing friction between particles [39].

The powder formulations are summarized in Table 1. Batches DR1, DR2, DR3, NDR1, NDR2 and DR4, were formulated with the same product ratio to obtain a reproducible formulation dosage.

P1, DR2, DR3, DR4 were dried at least 12 h at 20 °C in the Fisher Scientific Bio block oven (with 900 mbar vacuum and silica gel in the oven), as shown in Table 2. Batch NDR1 was directly extruded after grinding, and NDR2 was left in ambient humidity for at least 12 h. Batches DR2 and DR3 were dried for the same time (12 h) in order to check the repeatability of the process. Batch DR4 was dried for the same time as batches DR2 and DR3 in order to observe the influence of the extrusion parameters modification on the quality of the filament produced.

#### 2.2.2. Hot Melt Extrusion

The formulated powder was added into the force feeder (Thermo Fisher Scientific, Karlsruhe, Germany), which is a volumetric feeder at a monitored screw speed. This allows the powder to be drawn into the extruder. HME was carried out using a Pharma Mini HME at an extrusion temperature of 50 °C and 80 °C on the first and second half of the extruder. The system is a conical co-rotating twin-screw extruder with a rod-shaped aluminum die (Ø = 1.75 mm) (Thermo Fisher Scientific, Karlsruhe, Germany). The temperature of the production environment was 20 °C controlled by an Ebro, EBI 20 TH1 (Ebro, Freiburg im Breisgau, Germany. HME screw speed and force feeder speed were adapted depending on the batch produced, as shown in Table 3.

The filament diameter was controlled by a stericut-1 T (Citius engineering, Ougrée, Belgium), which carries out a regulation loop. Depending on the physical quality and the constituents of the formulation, the fluctuation around this target diameter is more or less important. It is, therefore, necessary to fully understand formulation properties that modify filament diameter. The diameter was adjusted with a 1.70 mm regulation by belt speed adjustment.

#### 2.2.3. Air Relative Humidity

In order to evaluate the environmental impact on powder processability, air humidity was measured with an Ebro, EBI 20 TH1 (Ebro, GE). Measurements were taken at the filament production zone, and an average value was recorded.

#### 2.2.4. Dynamic Vapor Sorption (DVS)

Water sorption and desorption of the formulation were evaluated by DVS measurement. A DVS resolution surface measurement system (SMS, London, UK) was used. About 10 mg of powder sample was added to the pan. DVS technology is a sensitive balance with a sample pan and an empty reference pan. The two pans were flushed with a controlled moist nitrogen stream. Samples were previously dried for 180 min at 0% RH at 25 °C by using a nitrogen stream. The step of humidity was set to 5% RH from 0 to 95% RH, then 95% RH to 0% RH (2 cycles). Anytime the control program detected a change in mass smaller than 0.002% per minute, the relative humidity changed automatically by 5% (max time per step 500 min).

#### 2.2.5. Mechanical Testing

**Figure 1:**
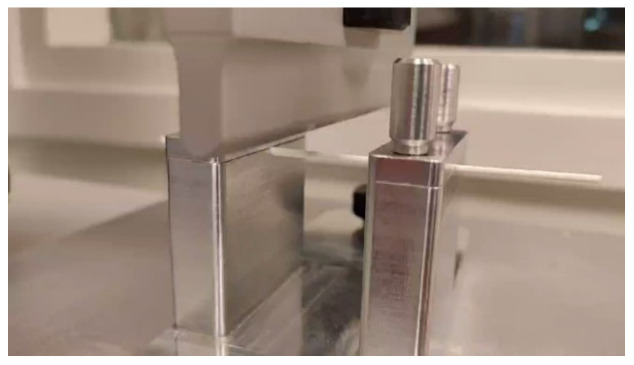
3 points bending setup with attached filament.

Filament mechanical properties were tested on a texture Analyzer TA-XT plus (Stable Micro Systems, London, UK) equipped with a 3-point bend rig HDP/3PB (Stable Micro Systems, London, UK), as shown in Figure 1 [40]. Testing was conducted with a blade speed of 3 mm/sec and a total displacement of 10 mm. The triggering force of the analysis is fixed at 0.25 N in order to limit the influence of weak forces, which can trigger the analysis at different distances depending on the sample orientation. Support spacing is 25 mm, and filaments are attached on either side of the support to allow easier reading of deformation profile and better results reproducibility. Tests were done in triplicate for all tested filaments. Three filament samples were cut to a length of 4 cm. The diameter of the filaments is regulated around 1.70 mm. Exponent version 4.0.13.0 software (stable micro Systems, London, UK) was used for data analysis and recovery.

#### 2.2.6. Thermal Analysis

Samples of raw materials, filaments and 3D-printed oral forms were characterized using a differential scanning calorimeter (DSC) Q2000 (TA instruments, Elstree, Hertfordshire, UK) with a heating rate of 10 °C/min [41]. Samples were heated with a single ramp from 20 °C to 190 °C. Analyses were carried out under a purge of nitrogen (50 mL/min). d-sorbitol peaks were integrated from 75 °C to 115 °C using TA 2000 universal analysis software (TA instruments, Hertfordshire, UK). Standard 40 µL TA aluminum pans and lids were used with an approximate sample mass of 5 mg.

For thermogravimetric analysis (TGA), samples of raw materials, filaments and 3D-printed oral forms were analyzed using a TGA Q500 (TA instruments, Hertfordshire, UK). Samples (10 mg) were placed in 40 µL aluminum pans and were scanned from ambient temperature to 300 °C at a heating rate of 10 °C/min [42]. The weight-loss profile was studied using TA 2000 universal analysis software (TA instruments, Hertfordshire, UK). Experiments were carried out under a nitrogen gas flow of 40 mL/min and 60 mL/min for sample and furnace, respectively.

#### 2.2.7. X-Ray Powder Diffraction (XRPD)

The powder X-ray diffraction analysis was carried out by a D8-Discover Bruker diffractometer with a copper anticathode tube with Kα radiation (λ = 1.540562 A) and Ni filter with a thickness of 0.5 mm. The voltage and current of the tube used are 40 KV and 40 mA, respectively. Samples were scanned over a 2-theta angle range from 2°to 40° with a step of 0.03° and a time per step of 0.5 s. The study of diffraction patterns was carried out using EVA version 5.1.0.5 software.

#### 2.2.8. Particle Size analysis Dry Dispersion Method (PSD)

Powder particle size was measured by laser diffraction (Mastersizer 3000, Malvern, GB, Malvern, UK) coupled with a dry dispersion unit without additional pressure in order to observe the PSD repartition of particles in the product.

#### 2.2.9. Scanning Electron Microscopy (SEM)

In order to assess the distribution and morphology of the various components in the mixtures, analysis was carried out using a scanning electron microscope (SEM) (JSM-IT 500HR, Jeol, Tokyo, Japan). Before any observation, the samples were placed on adhesive carbon tabs, themselves fixed on an aluminum specimen holder.

#### 2.2.10. Confocal Raman Microscopy

The distribution of components in the filament was analyzed by confocal Raman microscopy. The measurements were carried out with a WITec alpha 300 (Witec, Ulm, Germany. This system has a lateral resolution of 250 nm and a vertical resolution of 500 nm. The excitation wavelength is 532 nm. Mapping was carried out with an accumulation of 0.2 s and a resolution of 6 µm corresponding to a picture of 1500 × 1500 µm with 250 × 250 points. The acquisition is controlled using WITec Control software version 1.60. An entire Raman spectrum is thus collected per pixel. The spectra obtained were compared with the reference spectra obtained by the analysis of the individual components. Reference spectra were obtained with an accumulation time of 0.5 s and 100 accumulations. Measured spectra were compared to the spectra of the pure compounds to thus obtain a relative intensity of each component per pixel. From this data, an image is obtained representing the relative intensity of each compound. Data were processed using the WITec Project + software version 2.10 (Witec, Ulm, Germany).

#### 2.2.11. 3D Printing of the Dosage Forms

A cube of 10 mm side was modeled by computer using Openscad software version 2015.03. The design was then imported to the 3D printer’s Repetier software version 2.1.6.

FFF 3D printing was performed using a Prusa i3 Mk3S printer equipped with a 0.6 mm nozzle (Prusa Research, Prague, Czech Republic). Oral forms are placed on a steel tray with a smooth polyethylenimine coating supplied with the printer. Oral forms were printed using the settings presented in Table 4.

Once the slicing operation was performed, the length of the filament that was used by the printer, as indicated by the printer driver software. Three filament lengths (51, 100 and 200 mm) were imposed by modifying the dimensions of the object directly in the slicing software.

To avoid any cross-contamination issues, the 3D printer was dedicated throughout the duration of the study to the manufacture of amiodarone hydrochloride oral forms.

#### 2.2.12. Disintegration of Oral Forms in Syringe

The disintegration time of the oral forms was tested under conditions similar to ones done in hospitals. Therefore, each experiment was carried out on an oral form, which was put in a syringe containing 5 mL of water. The plunger was then added to the syringe, and manual agitation was performed. The complete disintegration time was recorded. Experiments were carried out in triplicate by the same manipulator.

## 3. Results

### 3.1. Powder Analysis

**Figure 2:**
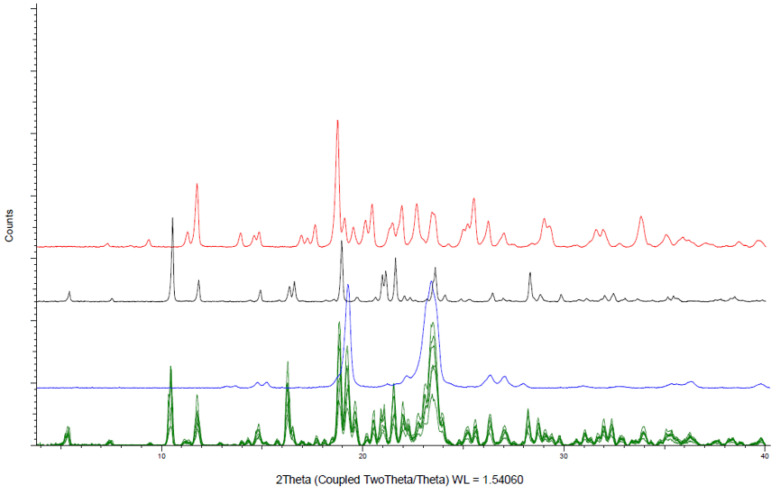
X-ray powder diffraction analysis of amiodarone hydrochloride formulations (**green**), amiodarone hydrochloride (**black**), PEO (**blue**), d-sorbitol (**red**).

XRD patterns of the six powder formulations shown in Figure 2 confirmed that amiodarone remains in the crystalline form indicated by a diffraction peak at 10.4° when mixed with other constituents. d-sorbitol and PEO retain their crystalline state after mixing identified mainly by diffraction peaks at, respectively, 19° and 23.5°. Colloidal anhydrous silica, as well as glycerol, were present in amounts too low to be identified by XRD.

**Figure 3:**
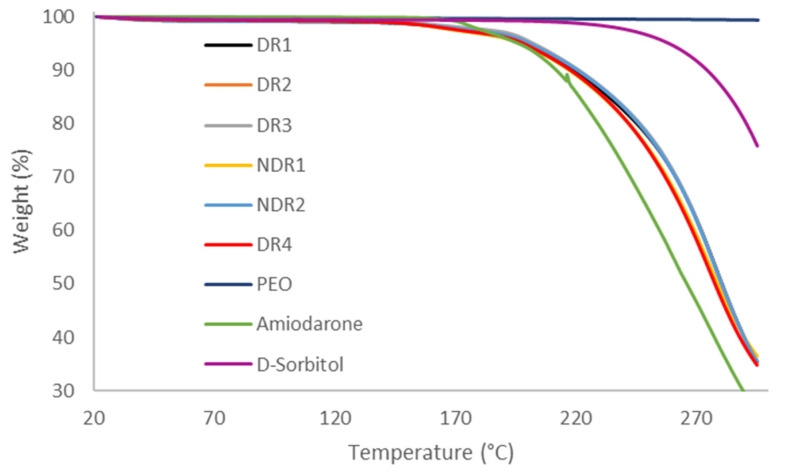
Thermal degradation profile of powders formulations before production.

In order to compare the thermal decomposition pattern and water absorption of the formulations in the state of powders, thermo-gravimetric analysis was carried out (Figure 3). Samples weight loss was integrated and reported in Table 5, revealing that all formulations lost around 1% of their weights after reaching 115 °C, probably due to the evaporation of adsorbed water. The degradation started at 160 °C due to amiodarone hydrochloride degradation. Therefore, no degradation was expected during HME and 3D printing because of a process temperature of 80 °C.

After hot-melt extrusion production, powders remaining into the hopper were analyzed by TGA and mass losses are reported in Table 5 in order to control a potential water absorption by the powder during the extrusion process. Water loss of powders into the hopper is the same as preproduction powders. TGA does not allow to observe differences in humidity between two samples at room temperature. In fact, before recording the mass loss data, samples were placed in the furnace of the instrument, under a flow of nitrogen then the data were recorded. Consequently, the measurement inertia does not make it possible to measure any change in the amount of surface water that can escape from the samples around room temperature.

**Figure 4:**
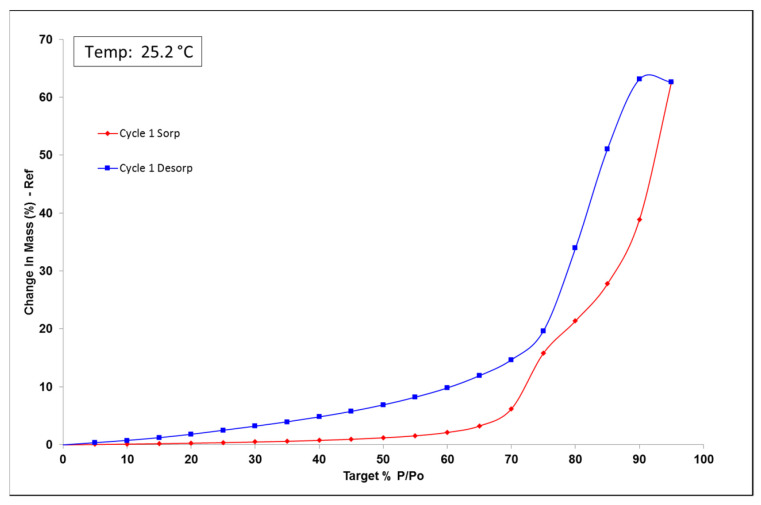
Amiodarone powder formulation dynamic vapor sorption (DVS) isotherm plot, sorption in red and desorption in blue.

The dynamic vapor sorption (DVS) analysis is a useful tool to observe powder water uptake comportment. The formulation was analyzed to see the impact of air humidity on powder change in mass. DVS data for the first cycle is reported in Figure 4. Up to 60% RH, formulation uptake was smaller than 5% RH indicating that the batch was non-hygroscopic. Beyond 60% RH, the formulation became deliquescent, indicated by plateau absence and water uptake of 60% because of d-sorbitol hygroscopicity [43,44]. Humidity environment can be an important parameter, and environment humidity should not be more than 60% RH. DVS isotherm plot indicates a hysteresis of water content between sorption and desorption. The hysteresis that was observed is related to the absorption kinetics. Indeed, the equilibrium was not reached after 500 min; the humidity was increased without the product having equilibrated with the medium. We, therefore, observed an absorption fault on the way up and an overestimation of absorption on the way down.

TGA analysis did not show variations in water adsorption depending on the powder storage. However, powder weighing was a useful tool to observe the change in the powder mass at ambient atmosphere. The weighing of the formulation before and after storage presented in Table 6 shown a 0.21% mass gain when the formulation was stored in an ambient atmosphere. Conversely, when the powder was stored in a dried environment, it lost 0.42% by mass. This simple analysis shows that water (not visible in TGA) is adsorbed by the powder in quantity depending on the storage.

**Figure 5:**
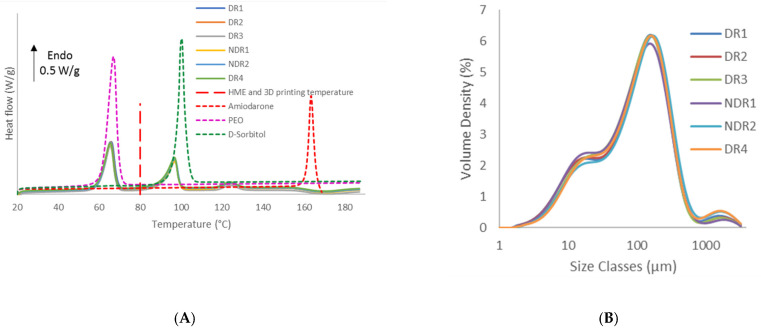
Differential scanning calorimeter (DSC) thermograms of amiodarone hydrochloride, polyethylene oxide (PEO), d-sorbitol and powders formulation (**A**) and particle size analysis dry dispersion method (PSD) graphs of the batches (**B**).

Thermal analysis data indicated that amiodarone hydrochloride, d-sorbitol and PEO remained in a crystalline form based on the presence of a melting peak, respectively, at 125 °C, 90 °C and 60 °C (Figure 5A). The melting peak of pure components indicated a melting point depression of amiodarone hydrochloride when mixed with other components. This phenomenon indicated interactions between the active ingredient and the other components [45,46]. The same DSC thermal scans also showed that all formulations presented the same thermal properties regardless of storage conditions. Regarding HME and 3D-printing temperature, it was expected a complete melting of PEO and a crystalline dispersion of d-sorbitol and amiodarone into the polymer matrix.

Grinding operation is influenced by factors, such as worker’s posture and motion. Variation of these parameters can influence the size and shape of particles [47]. PSD measurements (Figure 5B) showed a tri-modal distribution corresponding to individual products similar for each batch, respectively amiodarone hydrochloride, PEO and d-sorbitol. The dv_10_, dv_50_ and dv_90_ were, respectively, around 12 μm, 100 μm, and 320 μm for each batch analyzed, as shown in Table 7. As particle sizes were identical for all batches, storage conditions and grinding process do not impact particle size distribution of powders.

**Figure 6:**
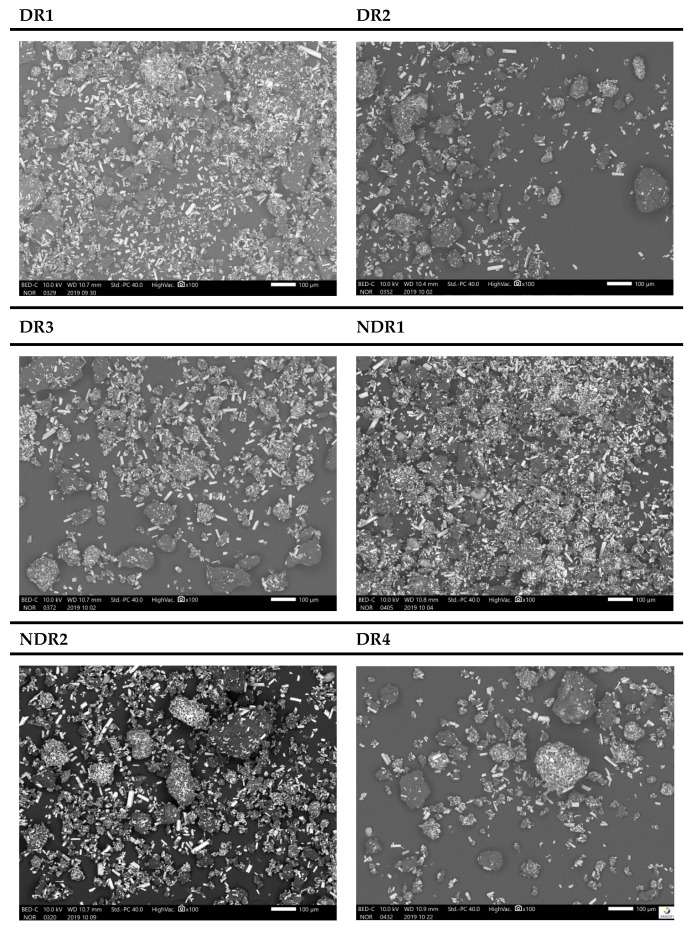
SEM micrographs with ×100 magnification of amiodarone formulations free powders after storage. From the top corner left to the bottom right are presented batches DR1, DR2, DR3, NDR1, NDR2 and DR4.

SEM micrographs (Figure 6) show columnar particles with lengths between 10 and 50 μm, which were identified as amiodarone because of their clarity (iodine atoms had a large atomic number) compared to other particles. Other particles were round or egg-shaped, and some particles had a maximum size of 150 μm. The morphology and repartition seemed identical in every batch due to the presence of amiodarone columnar particles as well as round or egg-shaped particles in every sample. SEM results did not highlight agglomerate formation or change in particle shape because of storage and grinding.

### 3.2. Filaments Characterization

**Figure 7:**
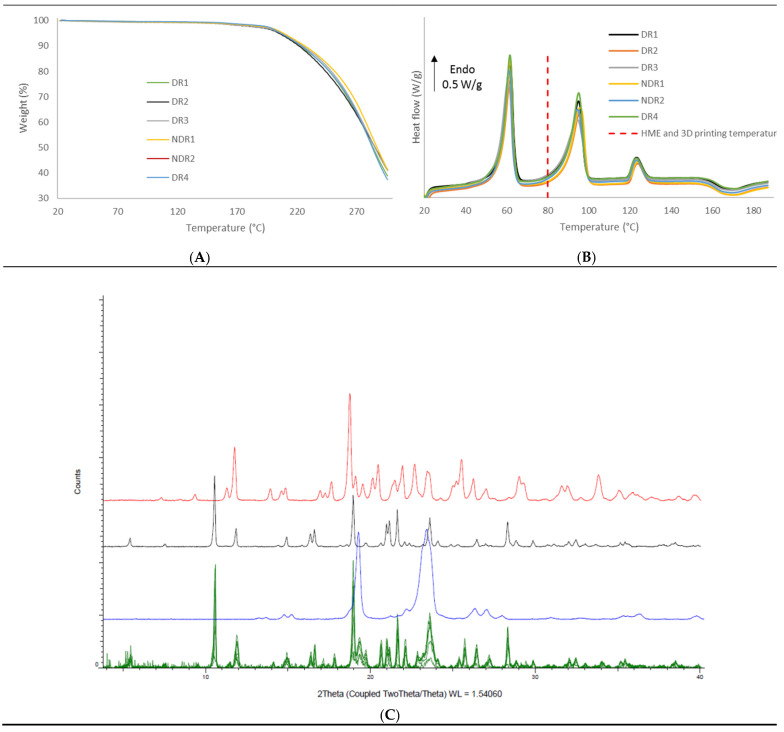
Thermal analysis and X-ray powder diffraction of PEO-based filaments. (**A**) Thermal degradation profile, (**B**) DSC thermograms of the six filaments batches and (**C**) X-ray powder diffraction spectra of the six filaments batches (green), PEO (purple), amiodarone hydrochloride (black) and d-sorbitol (red).

In order to compare the thermal decomposition pattern of the extruded filaments, thermo-gravimetric analysis was carried out (Figure 7A). No water absorption and absorption were observed, and filaments decomposition start at 160 °C. DSC thermograph showed (Figure 7B) that the melting point of amiodarone, PEO and d-sorbitol remained identical for all filament batches. The presence of such endothermic events in the DSC thermograph indicates that the majority of amiodarone and d-sorbitol existed in a crystalline form following HME. These results were confirmed by XRPD analysis (Figure 7C), where the spectra of the filament revealed diffraction peaks that match the diffraction pattern of amiodarone, d-sorbitol and PEO.

**Figure 8:**
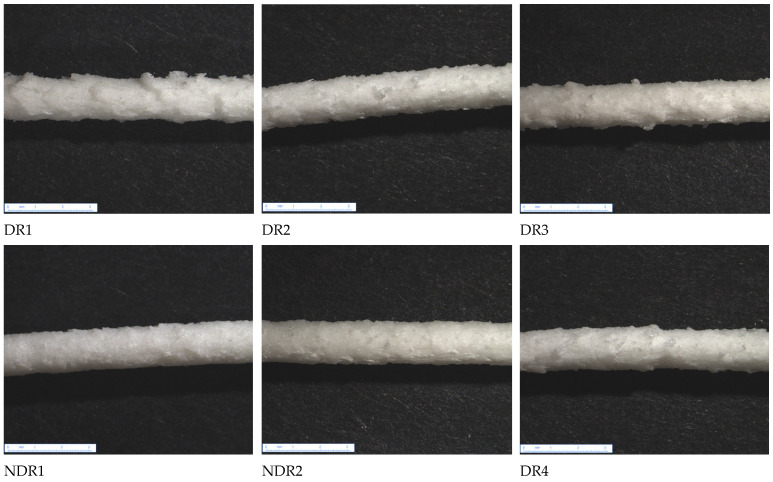
Amiodarone hydrochloride filaments under binocular microscope ×6.

Microscopy observations in Figure 8 showed a white filament surface due to a high quantity of crystalline and non-melted products present in the polymer matrix. Filaments presented a rough surface characteristic of the sharkskin phenomenon induced by high friction inside the die during HME [48]. However, surface smoothness was positively correlated with water content, as showed by weighing results Table 6. Indeed, filament NDR2 presented a smooth surface in comparison to other batches, as shown in Figure 8. Pereira et al. used water as a plasticizer for hot-melt extrusion [35], showing that water can decrease viscosity and friction during the HME process [32]. The water adsorbed by the powder, therefore, acts as a plasticizer during the HME process, making the extrudate more compact through the reduction in porosities. The more a powder has large amounts of water, the denser the extrudate is.

During filament production, in-process control allowed collecting different values of the filament diameter shown in Table 8. The filament maximum, minimum, average diameter, and relative standard deviation (RSD) of diameter were equivalent for all filaments produced by HME. This showed that regulation works correctly for all batches. Average belt speed was lower for NDR1 and NDR2 batches (5 mm/s versus 7 mm/s for the other batches). This value corresponded to the extrudate flow rate, which was linked to HME speed, force feeder speed, and powder flowability. However, from DR1 to NDR2 batches, extruder and force feeder speed were set constant at 25 and 3.5 RPM. Variation of average belt speed was the consequence of powder flowability modification.

It has been shown previously that the storage conditions modify the quantity of water contained in the powders. The lowest filaments rate of the regulation (and therefore, the lowest output rate) was observed for the non-dried powders (NDR1 and NDR2); these had the highest quantity of water. Indeed, the adsorbed water increased capillary interaction, which reduced the flow capacity of the powders [18,19]. This, therefore, explains storage conditions influence the regulation speed of the stericut system.

A change in flow rate can alter the physical characteristics of the filament produced. This is why a dried batch (DR4) was manufactured with a reduced extrusion speed in order to obtain an average belt speed of around 5.4 mm/s, which was identical to the non-dried batches (NDR1, NDR2). The objective was to compare the influence of HME flow on the apparent density and morphology of these batches.

The formulated powder can absorb a significant amount of water if the atmosphere is at a relative humidity above 60%. Therefore, we recorded atmosphere RH as presented in Table 9. The atmospheric RH was between 40 and 57%, which was lower than the critical value of 60% RH. During NDR1 production, atmosphere RH was 40%. However, this atmosphere relative humidity change did not impact change in flow or filament apparent density values.

A length of 200 mm for all filaments was sampled and weighed in order to observe linear mass density (LMD), as shown in Table 10. LMD of DR1, DR2, DR3 and DR4 batches was between 2.25 g/m and 2.38 g/m. The deviation from the mean value of the LMD obtained for these four batches was approximately 3%. The LMD was, therefore, equivalent and shown that it was possible to produce reproducible batches in terms of the filament LMD. The most dried batch (DR1) LMD seemed smaller than DR2, DR3 and DR4. Not dried batches (NDR1 and NDR2) presented LMD of 2.67 g/m and 2.76 g/m, which is more than 5% different from other LMD batch averages. The water present in the powder can act as a plasticizer, which can modify the surface state of the filaments and, therefore, impact the LMD. It highlights the fact that powder storage condition was an important parameter to obtain reproducible batches.

Batch DR4 was dried under the same conditions as batches DR2 and DR3. These three batches were identical, as shown in Table 10. However, the extrusion speed of batch DR4 was decreased to be produced at the same rate as batches NDR1 and NDR2. This proves that the water adsorption modified the extrusion speed, but the extrusion speed was not the parameter modifying the filament bulk density.

**Figure 9:**
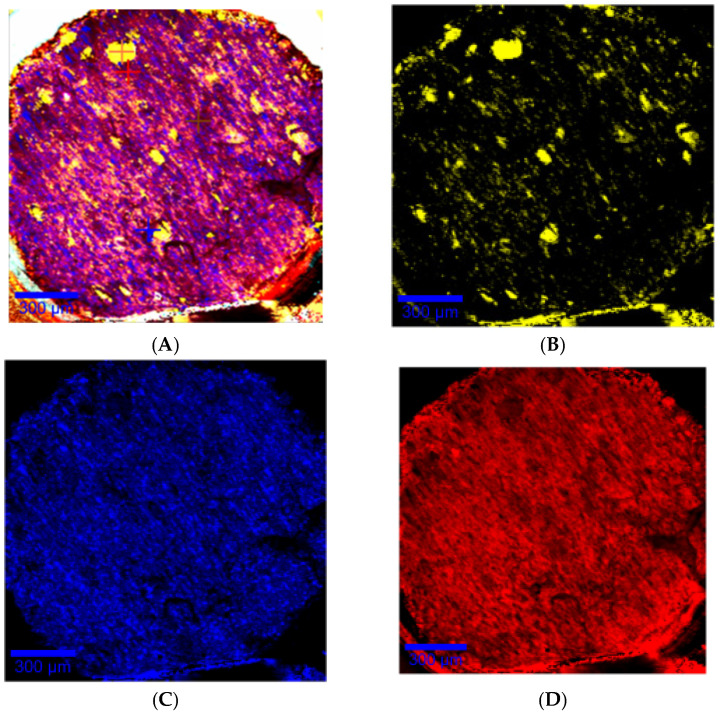
Raman mapping of the slice of a DR2 filament with the representative spectra of the individual compounds at the bottom (yellow: d-sorbitol; blue: amiodarone hydrochloride; red: PEO). Raman mapping is at the top with the same color code indicating the relative presence of the components of the filament with (**A**) all components, (**B**) d-sorbitol, (**C**) amiodarone hydrochloride, (**D**) PEO.

Confocal Raman mapping of the filament surface shown in Figure 9A exhibits repartition of components into the filament. Amiodarone and PEO were randomly and homogeneously distributed in the filament, as shown in Figure 9C,D). d-sorbitol was distributed in the form of particulate clusters of 20 to 100 µm characteristic of d-sorbitol particles introduced into the mixture, which were not melted, as shown in Figure 9B.

**Figure 10:**
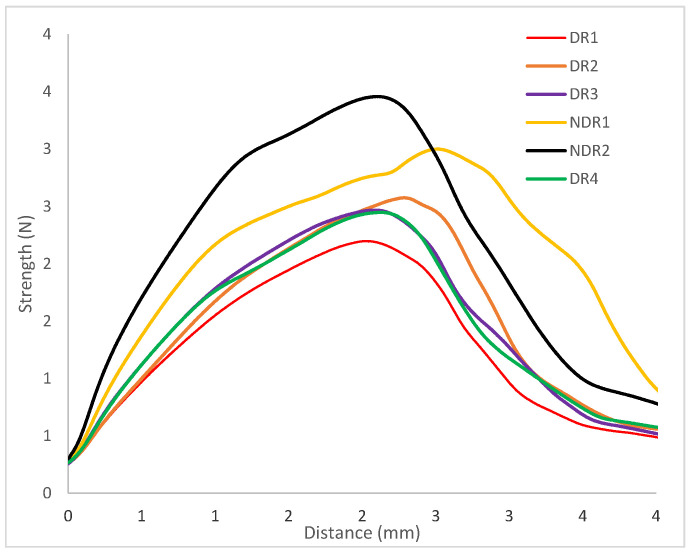
Load-deflection profiles of filament samples in tensile tests.

The mechanical behavior of the manufactured filaments is important in order to be able to manufacture oral forms by 3D printing. Three-point bending analysis of the filaments provided a load-deflection profile, as shown in Figure 10. All Samples exhibit elastic and ductile behavior. From the load-deflection profile, stiffness and elastic distance values were extracted and presented in Table 11. Elastic distances of the six filaments are equivalent, as shown by an elastic distance comprise between 0.92 mm and 1.13 mm. The filament elastic domain was identical whatever filament density and powder storage before HME production. Therefore, the surface condition and LMD of the filaments do not have a significant impact on their mechanical properties.

Filament stiffness was modified depending on the apparent density. Indeed, filaments DR1, DR2, DR3 and DR4 presented a stiffness between 1.2 N/mm and 1.4 N/mm. Therefore, filaments with equal apparent density presented the same stiffness. For filament NDR1 and NDR2, which were filaments with a bigger apparent density value, the stiffness coefficient was higher (respectively 1.8 N/mm and 2.1 N/mm). A three-point bending measurement allowed us to conclude that the elastic distance was not modified by storage condition contrary to the filament stiffness. Higher material density induced higher filament resistance to deformation.

### 3.3. Oral Forms Analysis

**Figure 11:**
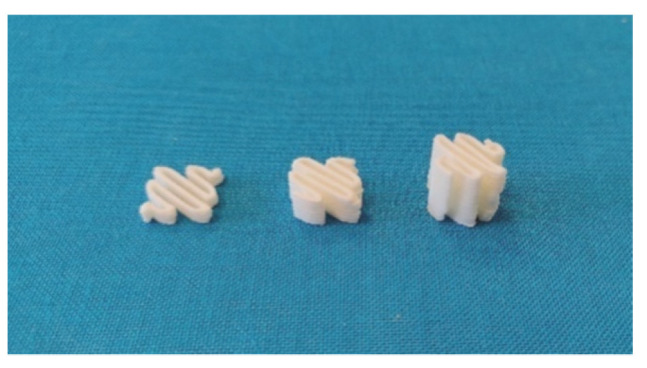
Photo of the three printed oral forms (from left to right, 51 mm, 100 mm and 200 mm filaments).

From filament produced by HME, 3D-printed oral amiodarone forms were compounded to be dispersed in 5 mL of water for administration to children. A defined filament length was used by the 3D printer to produce a defined dosage. The filament length was used as a reference value by the printer to make oral forms. Three filament lengths were selected, as shown in Figure 11, 51 mm, 100 mm, and 200 mm, with the aim of obtaining a calibration line surrounding a mass in oral form between 150 mg and 400 mg.

**Figure 12:**
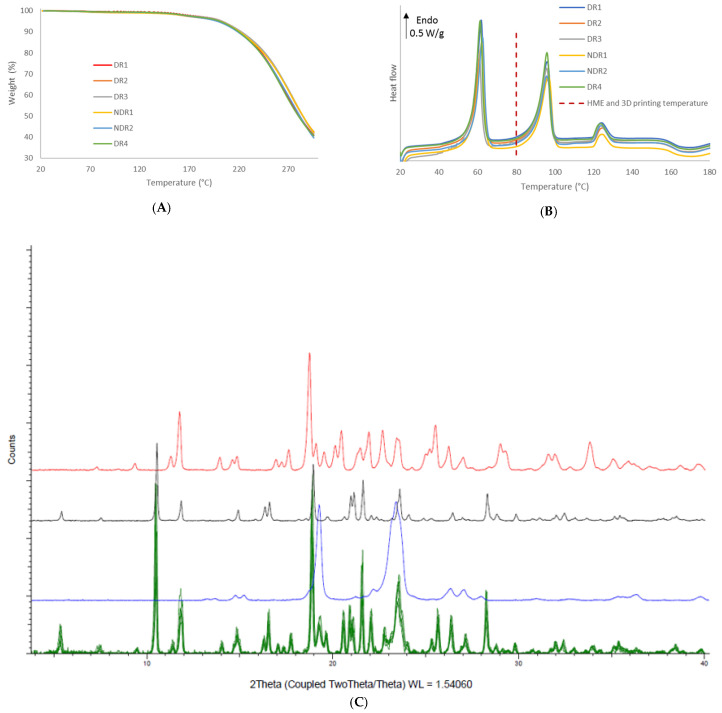
Thermal analysis and X-ray powder diffraction of PEO-based oral forms. (**A**) Thermal degradation profile, (**B**) DSC thermograms of the six oral forms batches and (**C**) X-ray powder diffraction spectra of the six oral forms batches (**green**), PEO (**blue**), amiodarone hydrochloride (**black**) and d-sorbitol (**red**).

In order to compare the thermal decomposition pattern of the oral forms, thermo-gravimetric analysis was carried out (Figure 12A). No water adsorption and absorption were observed. Oral forms decomposition had started at 160 °C, such as filaments and powders. DSC thermograph showed (Figure 12B) that the melting point of amiodarone, PEO and d-sorbitol remained identical for all filament batches. The presence of such peaks in the DSC thermograph indicated that the majority of amiodarone and d-sorbitol existed in a crystalline form following HME and the 3D-printing process. These results were confirmed by XRPD analysis (Figure 12C), where the oral forms spectra revealed diffraction peaks that match the diffraction pattern of amiodarone, d-sorbitol and PEO.

In order to produce a defined dosage, a calibration on the oral forms masses was made according to the filament length used by the printer. Some teams used the volume of the modeled object as a calibration tool [49]. However, the slicing operation could truncate parts of the volume according to the selected printing settings and, therefore, induced bias in oral form masses. Hence, the filament length was used as a reference value by the printer to define oral form masses. To do so, three objects with different sizes were printed, and the software provided the filament length needed. Manufactured objects were weighed, and a calibration curve was drawn in order to link the dosage to the filament length.

**Figure 13:**
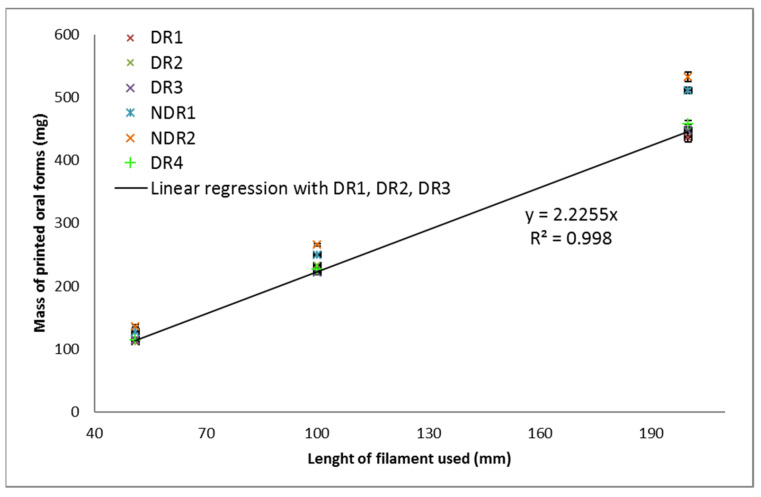
Oral form weights as a function of the filament quantity used by the 3D-printing process (**top**) and corresponding mass RSD (**bottom**).

Within the same filament batch, the oral form mass relative standard deviation was smaller than 5%, as shown in Table 12. The oral forms made from batches DR1, DR2, DR3 and DR4 were equivalent in terms of masses, as shown in Figure 13. These batches were dried under similar conditions, which highlights the importance of this step for the production of constant oral forms. NDR1 and NDR2 filaments induced the production of oral forms with larger masses than the other batches. These batches were not dried before HME production, which shown that the drying operation reduced the mass of the oral forms produced. Linear regression was carried out on the mass of the oral form produced from batches DR1, DR2, DR3, which are produced in similar 3D-printed and HME conditions. The leading coefficient was 2.2, and the coefficient of determination was 0.999 indicating the reliability of linear regression. The leading coefficient can, therefore, be used to determine the filament length to be used by the printer to produce a given oral form mass.

From the results presented in Figure 13, linear regressions were carried out for all batches, and the corresponding leading coefficients were obtained. Oral form masses could be determined by multiplying the leading coefficient by the length of the filament that was used by the printer to produce a given object. The leading coefficient obtained for each batch was compared as presented. The percentage obtained corresponds to the mass difference between the two batches. Percentages greater than 5% did not allow reproducible mass production according to the pharmacopeia criteria regarding mass uniformity [9]. These values were, therefore, underlined in red. Results highlighted that batch DR1, DR2, DR3 and DR4 were similar in terms of the leading coefficient. The greatest difference in coefficient was between batches DR1 and DR4 (4%). The lowest coefficient difference was 1% between batches DR2 and DR3, which were prepared identically. Despite the fact that these filaments were produced separately, they allowed the manufacture of oral forms with similar masses. Batches NDR1 and NDR2, which were not dried shown a large difference in coefficient with the other batches (more than 10%), indicating that oral forms masses were distant.

Oral forms were dispersed in 5 mL of water to observe the impact of powder storage conditions on the oral forms’ disintegration time, as shown in Table 13. Regardless of the batch analyzed, the disintegration time was 3 min when oral forms are made with 51 mm of the filament, 4 min with 100 mm and 5 min with 200 mm of the filament. The volume of water that was used was the same for all three assays. The oral forms disintegration mechanism was erosion because of high thermoplastic polymer quantity [50]. Therefore, the greater the mass of the oral forms, the greater the disintegration time. Results have demonstrated that it was possible to produce rapidly disintegrating oral forms in addition to child food.

Batches DR2 and DR3 were manufactured under the same conditions. This is why the leading coefficient resulting from the calibration of these two batches was used to produce 300 mg oral forms containing 60 mg of amiodarone hydrochloride. The average leading coefficient is 2.24415. To produce 300 mg oral forms, the modeled object should be made from 133.7 mm of filaments. From this object, 20 oral forms were manufactured and weighed, as shown in Table 14, in order to carry out a mass uniformity control according to European Pharmacopoeia 10.2 recommendations [9]. The 20 oral forms samples average mass was 301.80 mg. The target mass was 300 mg, so the measured average mass was deviated by 0.6%. The mass RSD was 1.7%, demonstrating the reproducibility of the 3D-printing process. In addition, none of the weighed oral forms have a mass outside the percentage deviation required by pharmacopeia.

From a filament containing 20% of amiodarone hydrochloride, it was possible to carry out a calibration by manufacturing oral forms by 3D printing in order to correlate the quantity of the filament used by the printer according to the oral form mass. Thus, it is possible to model with a precise dosage of oral forms in advance, making it possible to know the mass and, therefore, the dosage of the oral forms before printing.

**Figure 14:**
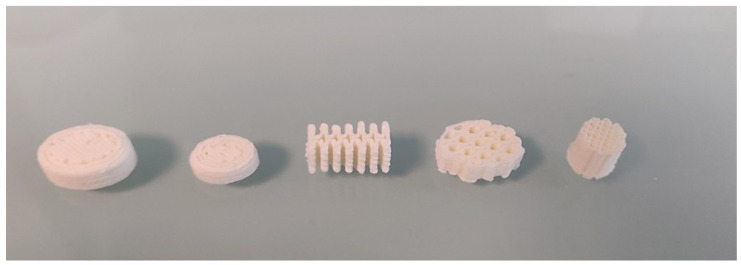
Photo of oral amiodarone forms showing different shapes and sizes.

The oral forms presented above are only a superposition of identical layers. In order to demonstrate the possibility of producing oral forms with complex geometry, we fabricated oral forms with various geometries, as shown in Figure 14. This example shows that it is possible to produce oral amiodarone forms with a geometry adapted to patient’s needs.

## 4. Conclusions

To produce antiarrhythmic personalized oral forms, fast disintegration oral forms were produced by 3D-printing technology. To better understand and master this new way of producing oral forms, batch reproducibility was studied and reported in this work.

Every batch is allowed to produce oral forms with a mass percentage variation smaller than 5%. However, from a batch to another, oral forms masses varied. Powder formulation storage before the HME process was identified as an important parameter to control HME feed rate, filament LMD and, therefore, oral forms masses.

It was demonstrated that amiodarone formulations adsorbed ambient water, which changes powder flow properties even when the relative humidity of the atmosphere was less than 60%. In addition, the water acted as a plasticizer with a percentage as low as 1% in mass in the hot extrusion process, which changed filaments LMD by modifying the surface roughness of the filaments.

By controlling the drying time, it was possible to produce equivalent filament batches. From two batches produced under identical conditions, it was possible to carry out a calibration, making it possible to predict the corresponding oral form mass from a modeled object. A batch of 20 units of 300 mg oral forms was made. This batch complied with European pharmacopeia in terms of mass uniformity, demonstrating that 3D printing by deposition of molten material can allow manufacturing reproducible and precise batches. The filament quality master is essential for considering oral form compounding for personalized medicine in healthcare establishments, as close as possible to the patient.

## Figures and Tables

**Table 1:** Summary of the mass percentage of each product for all formulations.

Product	Mass Percentage (%)
PEO	40%
d-sorbitol	37%
Glycerol	2%
SiO_2_	1%
Amiodarone hydrochloride	20%

**Table 2:** Batch storage conditions before filament production.

Batch	Storage Conditions
DR1	Dried 48 h
DR2	Dried 12 h
DR3	Dried 12 h
NDR1	Dried 0 h
NDR2	Ambient humidity 12 h (50% RH)
DR4	Dried 12 h

**Table 3:** Powder storage conditions before extrusion and hot-melt extrusion (HME) parameters in function of the batch.

Batch	HME Screw Speed (RPM)	Force Feeder Speed (RPM)
DR1	25	3.5
DR2	25	3.5
DR3	25	3.5
NDR1	25	3.5
NDR2	25	3.5
DR4	23	2.7

**Table 4:** 3D-printing slicing settings.

Parameter	Value
Layer heights	0.4 mm
Nozzle temperature	80 °C
Bed temperature	30 °C
Number of top and bottom layers	0
Number of perimeters	0
Infill	40%
Infill pattern	Rectilinear
Speed for print moves	10 mm/s
Speed for non-print moves	120 mm/s

**Table 5:** Summary of mass losses in thermogravimetric analysis (TGA) from ambient temperature to 115 °C.

Batches	Mass Loss (%)	
	Before Production	Powder that Remained in the Hopper after Production
DR1	0.95	0.91
DR2	0.83	1.00
DR3	0.81	0.84
NDR1	0.77	0.91
NDR2	0.92	1.18
DR4	0.79	0.75

**Table 6:** Mass variation of the powder formulation depending on storage.

Powder Storage Condition	Initial Mass (g)	Mass after 24 h (g)	Mass Variation (%)
Dried	8.8950	8.8580	−0.42%
Ambient humidity (40% HR)	10.4933	10.5153	0.21%

**Table 7:** Summary table of powders mixtures PSD analysis.

Batch	Dv_10_ (μm)	Dv_50_ (μm)	Dv_9 0_(μm)
DR1	11.9	102	312
DR2	12.0	102	315
DR3	13.3	100	305
NDR1	12.1	95.5	305
NDR2	13.8	115	363
DR4	14.1	107	340

**Table 8:** Summary table of stericut analysis.

Parameter	DR1	DR2	DR3	NDR1	NDR2	DR4
Average diameter (mm)	1.701	1.701	1.701	1.701	1.703	1.700
Diameter standard deviation (mm)	0.026	0.021	0.021	0.019	0.037	0.022
Diameter RSD (%)	1.52%	1.20%	1.26%	1.10%	2.20%	1.32%
Maximum (mm)	1.803	1.788	1.783	1.754	1.804	1.764
Minimum (mm)	1.632	1.636	1.638	1.654	1.625	1.630
Number of measurement points	1600	1200	1400	500	500	1200
Average belt speed (mm/s)	6.9	6.8	7.0	5.4	5.0	4.92

**Table 9:** Summary table of atmosphere relative humidity during HME production.

Batch	Atmosphere RH (%)
DR1	55%
DR2	57%
DR3	56%
NDR1	40%
NDR2	50%
DR4	49%

**Table 10:** Summary results of filaments linear mass density (LMD).

Parameter	Batch					
	DR1	DR2	DR3	NDR1	NDR2	DR4
Filament LMD (g/m)	2.25	2.31	2.36	2.66	2.75	2.38

**Table 11:** Summary of texturometer analysis.

Parameter	Batch					
	DR1	DR2	DR3	NDR1	NDR2	DR4
Stiffness (N/mm)	1.19 ± 0.07	1.36 ± 0.10	1.44 ± 0.10	1.83 ± 0.28	2.08 ± 0.22	1.45 ± 0.10
Elastic distance (mm)	1.06 ± 0.08	1.14 ± 0.00	0.97 ± 0.02	0.92 ± 0.05	0.98 ± 0.12	0.93 ± 0.08

**Table 12:** Mass relative standard deviation of batches according to the printed filament length.

Filament Length (mm)	Mass Relative Standard Deviation (%)					
	DR1	DR2	DR3	NDR1	NDR2	DR4
51	1.1%	3.2%	1.6%	0.6%	1.3%	0.2%
100	0.7%	1.0%	1.6%	0.4%	0.6%	0.6%
200	1.6%	0.7%	2.0%	1.0%	1.4%	1.1%

**Table 13:** Oral form disintegration times for three 3D-printed dosages.

Filament Length Used	Disintegration Time for All Batches (min)
51 mm	3
100 mm	4
200 mm	5

**Table 14:** Mass, mass relative standard deviation (RSD) and number of samples falling outside pharmacopeia standards based on mass uniformity results of oral form masses printed from 113.7 mm of the filament.

Parameter	Value
Average (mg)	301.80
Minimum (mg)	292.24
Maximum (mg)	310.47
Percentage deviation (%)	1.7%
5% deviation maximum mass (mg)	315
5% deviation minimum mass (mg)	285
10% deviation maximum mass (mg)	330
10% deviation minimum mass (mg)	270
Samples outside 5% deviation	0
Samples outside 10% deviation	0
